# An Investigation on the Spark Plasma Sintering Diffusion Bonding of Diamond/Cu Composites with a Cr Interlayer

**DOI:** 10.3390/ma17246026

**Published:** 2024-12-10

**Authors:** Ying Zhou, Daochun Hu, Minghe Chen, Taowen Wu, Jindong Ouyang, Degan Xiong

**Affiliations:** 1 College of Mechanical & Electrical Engineering, Nanjing University of Aeronautics and Astronautics, Nanjing 210016, China; zzzying@nuaa.edu.cn (Y.Z.); meemhchen@nuaa.edu.cn (M.C.); meewtw0116@nuaa.edu.cn (T.W.); 2 School of Mechanical Engineering, Nanjing Vocational University of Industry Technology, Nanjing 210023, China; 3 Jiangxi Hongdu Aviation Industry Group Co., Ltd., Nanchang 330024, China; daohewei15@nuaa.edu.cn; 4 Hunan Province Engineering Research Center for High Thermal Conductivity Metal-Matrix Composites, Hunan Harvest Technology Development Co., Changsha 410219, China; lx210103@sina.com

**Keywords:** diamond/Cu composites, SPS diffusion bonding, Cr interlayer, thermal conductivity

## Abstract

Spark plasma sintering (SPS) is an effective technique for studying the diffusion bonding of diamond/Cu composites, and has the potential to advance the application of copper matrix composites. This study investigates the SPS diffusion bonding of diamond/Cu composites using a chromium (Cr) interlayer. The effects of process parameters on the microstructure and mechanical properties of the bonding interface were evaluated through shear strength testing and SEM analysis. The results show that shear strength increases with interlayer thickness up to a certain point, after which it decreases. As the bonding temperature, holding time, and bonding pressure increase, defects such as cracks and voids at the diffusion-bonded interface are reduced, resulting in improved shear strength. Under suitable conditions (10 μm interlayer, 810 °C, 60 min, and 10 MPa), the bonding interface is defect-free, achieving a maximum shear strength of 139.89 MPa and a thermal conductivity (TC) of 700.97 W/(m·K), indicating high-quality diffusion bonding.

## 1. Introduction

With the rapid advancement of microelectronic technology, electronic components are increasingly being miniaturized and integrated with more functions. As a result, the heat flow density in microelectronic chips has risen dramatically [1,2], leading to significant reductions in chip lifespan and reliability. Efficient heat dissipation in microelectronic chips has become a critical issue [3,4]. Diamond/Cu composites, which combine high thermal conductivity (TC) with a low coefficient of thermal expansion, have emerged as a promising solution for heat dissipation [5,6]. Most existing studies have focused on improving the preparation of diamond/Cu composites to address the weak chemical affinity between Cu and diamond. However, diamond’s difficulty in being processed into complex shapes, combined with current processing methods that are limited to producing simple geometries, restricts the application of diamond/Cu composites [7,8,9].

Brazing is considered the most promising process for fabricating complex shapes. However, the chemical properties of diamond differ significantly from those of common brazing fillers, making it difficult for brazing fillers to wet the diamond surface. Moreover, most brazing fillers are brazed at temperatures above 800 °C. Higher brazing temperatures result in higher residual stresses in the joint [10]. Diffusion bonding can produce weldments with extremely complex shapes, and the joints have the same properties as the base metal [11]. However, traditional diffusion bonding requires higher temperatures and longer heating times for overall heating in the furnace. Prolonged exposure to high temperatures can lead to the graphitization of the diamond surface, which severely reduces its ability to dissipate heat [12].

Spark plasma sintering (SPS) diffusion bonding is an advanced bonding technique that uses a pulsed current to generate plasma discharge, which uniformly produces Joule heat in each component, enabling perfect joining under applied pressure [13]. Ananthakumar et al. [14] investigated the SPS diffusion bonding of austenitic stainless steel (AISI 304L) and titanium, achieving a superior shear strength of 429 MPa at 650 °C. Pan et al. [15] examined the diffusion bonding of different steels with varying Ni interlayer thicknesses, obtaining a maximum bending strength of 982 MPa. Li et al. [16] achieved a joint strength of 271 MPa in their study on Cu-Cu SPS diffusion bonding. SPS offers advantages such as rapid heating, low energy consumption, and the ability to maintain low temperatures [17], thereby preserving the properties of the base materials and ensuring reliable bonding. However, due to the weak chemical affinity between Cu and diamond, cracks, voids, and other defects may form at the bonding interface, which can adversely affect heat transfer efficiency [18,19,20]. To obtain high-quality joints, it is often necessary to insert an interlayer at the bonding interface [21]. Chromium (Cr) exhibits a strong chemical affinity for carbon, enabling the formation of stable carbides at the interface. These carbides play a crucial role in enhancing the interfacial bonding between Cu and diamond, which, in turn, improves the thermal performance of the composites [22,23,24,25,26,27].

In this research, diamond/Cu composites were bonded via the SPS diffusion bonding method with a Cr interlayer. The research investigated the impact of bonding temperature, the thickness of Cr foil, holding time, and bonding pressure on both the properties and microstructure.

## 2. Materials and Methods

### 2.1. Materials

**Figure 1:**
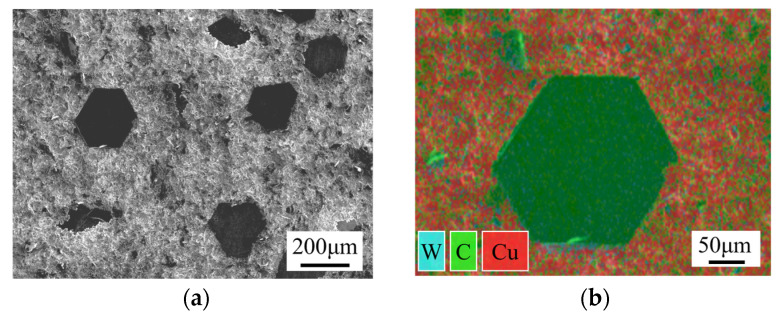
Microstructure and element distribution of diamond/Cu composites: (**a**) micro-morphology; (**b**) element distribution.

The base material for the SPS diffusion bonding experiment was diamond/Cu composites, and the interlayer was a foil with 99.99% Cr content. The diamond/Cu composites were prepared as follows: First, tungsten (W) with a thickness of 100 nm was deposited on the surface of the diamond through magnetron sputtering to metallize it. Then, diamond/Cu billets were fabricated using the vacuum pressure infiltration method. Diamond constituted about 60% of the volume of the composites. The TC of the diamond/Cu composites was 818.67 W/(m·K). Figure 1 illustrates the microstructure and element distribution of the diamond/Cu composites.

### 2.2. SPS Diffusion Bonding Experiments

**Figure 2:**
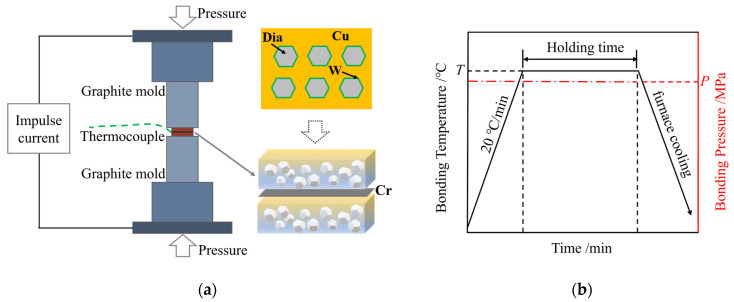
(**a**) Schematic diagram of SPS diffusion bonding; (**b**) temperature/pressure/time curves.

The experimental materials were stacked sequentially and placed in the spark plasma sintering furnace (Threetimes Equipment Co., Ltd., Shenzhen, China) for joining, as depicted in Figure 2a. The SPS system (Threetimes Equipment Co., Ltd., Shenzhen, China) has a maximum output current of 30,000 A and a pulse frequency range of 5 to 5000 Hz. a temperature/pressure/time curve of the diffusion bonding process is shown in Figure 2b. The heating rate was 20 °C/min, the cooling method was furnace cooling, and the pulse duty cycle was set to 10 ms:10 ms. SPS diffusion bonding requires an appropriate range of process parameters for experimentation. The bonding temperature is typically 0.6 to 0.8 times the melting point of the material. Additionally, the atomic diffusion requires a suitable holding time and bonding pressure. Based on preliminary experimental exploration, the process parameters were determined to fall within the following ranges: an interlayer thickness of 10~50 μm, a bonding temperature of 720~810 °C, a holding time of 30~90 min, and a bonding pressure of 7.5–12.5 MPa. The details of the experimental design are presented in Table 1.

### 2.3. Characterization and Analysis

The diamond/Cu composites after SPS diffusion bonding were laser-cut. The microstructure of the bonded interface was observed using a Sigma300 scanning electron microscope (SEM) (ZEISS Group, Oberkochen, Germany). The content and distribution of elements on the bonded interface were measured using an Xplore30 energy-dispersive spectrometer (EDS) (BEST Instrument Equipment Co., Ltd., Shenzhen, China). The phase of the diffusion bonding interface was analyzed using a SmartLab 9 kW X-ray diffractometer (XRD) (Bright Industrial Co., Ltd., Shanghai, China).

**Figure 3:**
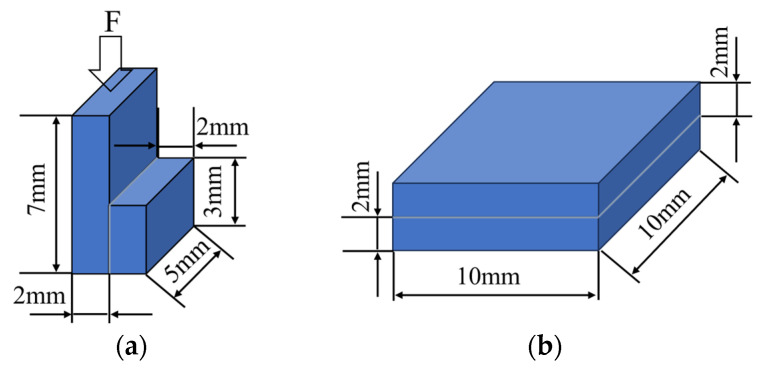
A schematic illustration of (**a**) the shear test and (**b**) the sample for the TC test.

To evaluate the quality of the SPS diffusion bonding, the shear strength of the bonded pieces was tested at room temperature using a UMT5000 universal tensile test machine. (SUNS Technology Co., Ltd., Shenzhen, China) A schematic illustration of the shear test is shown in Figure 3a.

The thickness of the samples was measured with a micrometer screw (QLR Co., Ltd., Changzhou, China) before and after SPS diffusion bonding. The average thickness of the sample was taken from five points. The deformation rate of the joint is given by

*δ* = (*h*_0_ − *h*_1_)/*h*_0_ (1)

where *δ* is the deformation rate of the joints (%), *h*_0_ is the thickness of the samples before diffusion bonding (mm), and *h*_1_ is the thickness of the samples after diffusion bonding (mm).

The joints were laser-cut into blocks, as shown in Figure 3b, and the thermal diffusion coefficient was measured via the laser flash method using an LFA 467HT instrument (QLR Co., Ltd., Changzhou, China). The TC was calculated as follows:

*λ = αρC* (2)

where *λ* is the TC of the composites (W/(m∙K)), *α* is the thermal diffusion coefficient (mm^2^/s), *ρ* is the density of the diamond/Cu composite (g/cm^3^), and *C* is the heat capacity of the composites (J/(g∙K)).

## 3. Results and Discussion

### 3.1. The Effect of the Thickness of the Interlayer

**Figure 4:**
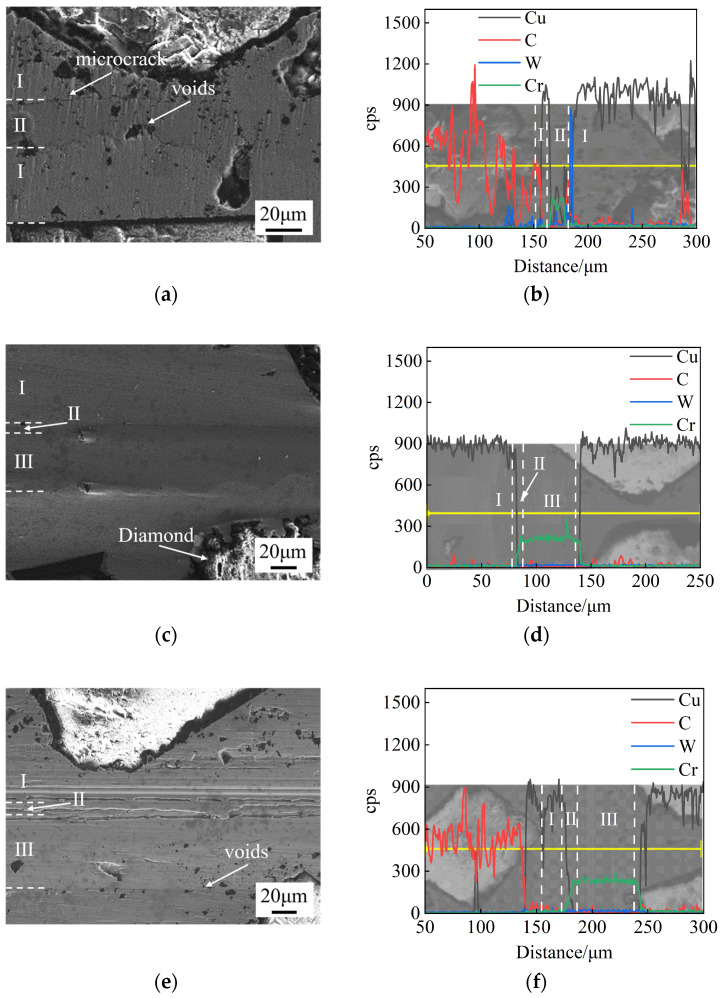
Microstructure and EDS results of bonding interface at 750 °C, 60 min, and 10 MPa for different interlayer thicknesses: (**a**,**b**) 10 μm; (**c**,**d**) 30 μm; (**e**,**f**) 50 μm.

Cr foils with different thicknesses (10 μm, 30 μm, 50 μm) were used as the interlayer for SPS diffusion bonding at 750 °C for 60 min under a pressure of 10 MPa. Figure 4 shows the microstructure and EDS results of the bonding interface for different foil thicknesses. The diffusion bonding interface consists of three characteristic layers: the Cu matrix (I), the diffusion layer (II), and the undiffused interlayer (III).

When the foil thickness is 10 μm, microcracks and voids are observed at the bonding interface, as shown in Figure 4a. Significant diffusion occurs between Cu and Cr, with some Cu atoms migrating through the interlayer into the Cu matrix region (I) on both sides. The thickness of the Cu-Cr diffusion layer (II) is approximately 16 μm, as shown in Figure 4b. When the foil thickness is 30 μm, no obvious defects are observed at the bonding interface, and the joint is well formed (Figure 4c). The thickness of the diffusion layer (II) is approximately 7 μm. When the foil thickness is 50 μm, discontinuous voids are observed at the bonding interface. The thickness of the diffusion layer (II) is approximately 9 μm, as shown in Figure 4e,f.

The shear strength and deformation rate for different interlayer thicknesses are shown in Table 2. As the thickness increases from 10 μm to 30 μm, the shear strength rises from 108.97 MPa to 131.27 MPa. However, when the thickness increases from 30 μm to 50 μm, the shear strength decreases to 63.60 MPa due to the formation of cracks and voids. Additionally, as the interlayer thickness increases, the deformation rate increases from 0.78% to 1.16%.

### 3.2. Effect of Bonding Temperature

**Figure 5:**
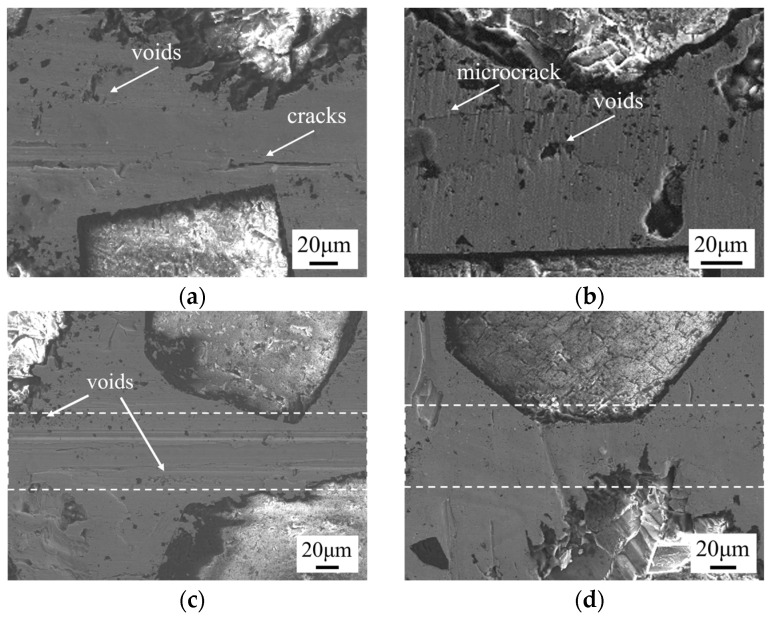
Microstructure of bonding interface at different temperatures under 10 μm, 60 min, and 10 MPa: (**a**) 720 °C; (**b**) 750 °C; (**c**) 780 °C; (**d**) 810 °C.

Bonding temperature is a key parameter affecting the performance of SPS diffusion bonding [28]. The microstructure of the bonding interface at different temperatures (720 °C, 750 °C, 780 °C, 810 °C) for a foil thickness of 10 μm, a holding time of 60 min, and a pressure of 10 MPa is shown in Figure 5. At a bonding temperature of 720 °C (Figure 5a), obvious cracks and continuously distributed voids are observed at the bonding interface. This is because, during the SPS diffusion bonding process, the heat source is generated by the Joule heating from the pulse current passing through the sample. At lower bonding temperatures, the pulse current is smaller, resulting in lower atomic diffusion ability. At 750 °C (Figure 5b), voids and microcracks are observed at the interface. As the temperature increases to 780 °C (Figure 5c), the cracks at the bonding interface disappear and fewer voids are present. At 810 °C (Figure 5d), no significant defects are observed at the bonding interface, and the joint formation is excellent, indicating that higher bonding temperatures promote better interfacial bonding and reduce defect formation.

**Figure 6:**
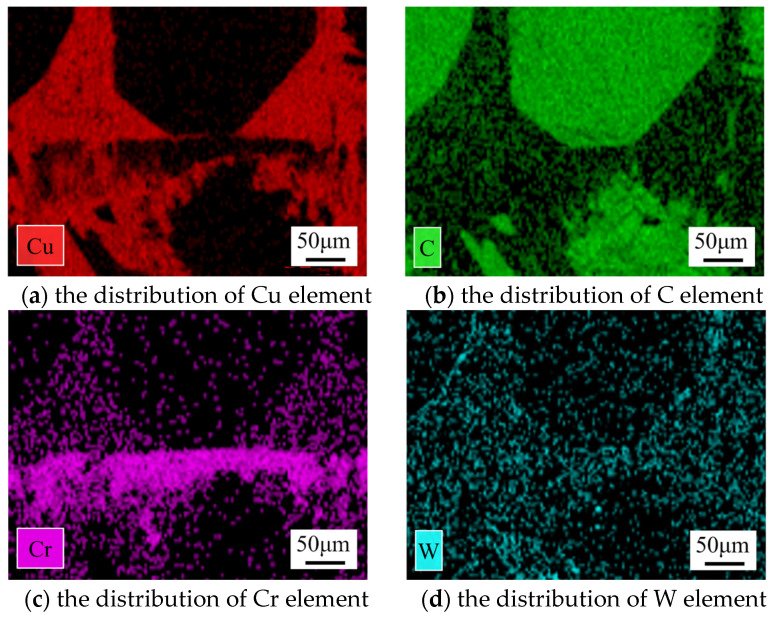
EDS analysis of bonding interface at 810 °C.

**Figure 7:**
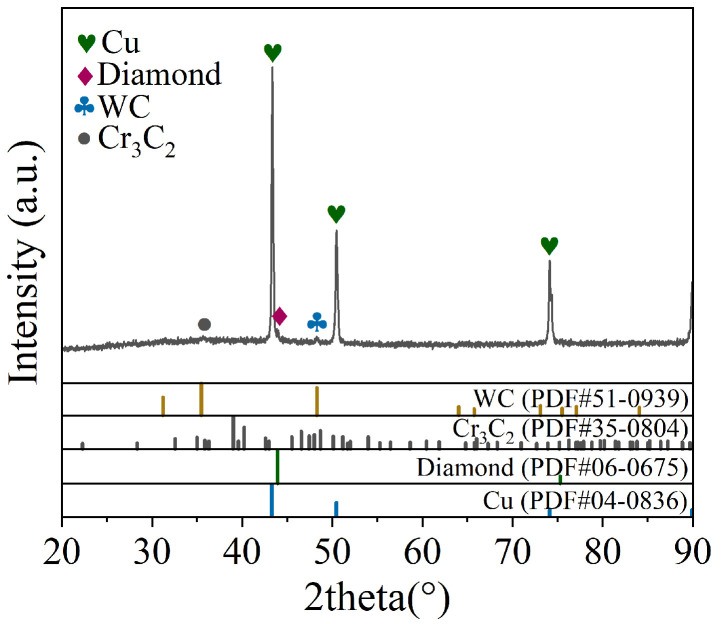
XRD patterns of bonding interface at 810 °C.

Figure 6 shows the EDS analysis of the bonding interface at 810 °C, indicating that Cr diffuses into the copper matrix and the surface of the diamond, while C diffuses into the copper matrix and the Cr interlayer. The overlapping regions of metallic elements and C suggest the potential formation of carbides. Figure 7 shows the XRD pattern of the bonding interface at 810 °C. Diffraction peaks of Cu, diamond, WC, and Cr_3_C_2_ phases are observed at the bonding interface, indicating that Cr atoms in the interlayer react with C atoms in the diamond to form the Cr_3_C_2_ phase. Cr_3_C_2_ can form at relatively low temperatures [29], which may be attributed to two factors: on the one hand, the high current density in the SPS process reduces the activation energy required for the formation of Cr_3_C_2_ carbide, thereby facilitating the reaction; on the other hand, the electric current promotes the migration of charged ions, accelerating the reaction of metal atoms and carbon atoms, leading to the synthesis of Cr_3_C_2_.

Table 3 shows the shear strength and deformation rate of the joints at different bonding temperatures. As the bonding temperature increases from 720 °C to 810 °C, both the shear strength and deformation rate increase. At 810 °C, the shear strength reaches 139.89 MPa, nearly double that at 720 °C, while the deformation rate is 1.09%. This improvement is primarily due to enhanced atomic diffusion during SPS diffusion bonding, which promotes the formation of Cr_3_C_2_ at the interface. The bonding interface changes from mechanical to chemical bonding, which increases the bond strength. Additionally, higher temperatures enhance the plastic deformation capability of the copper matrix. The areas of close contact transition from localized point contact to more extensive surface contact, gradually eliminating cracks and voids between the interfaces, resulting in a stronger bonded interface.

### 3.3. Effect of Holding Time

**Figure 8:**
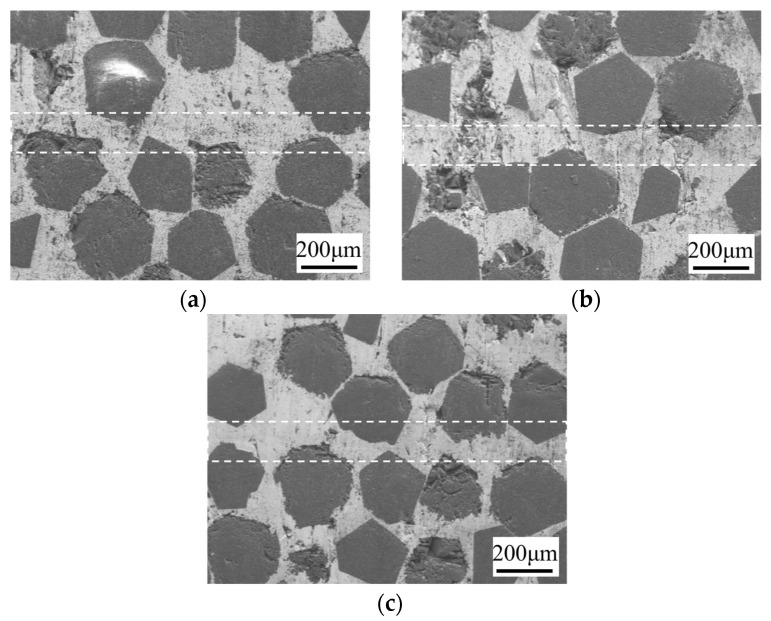
Microstructure of bonding interface at different holding times under 10 μm, 750 °C, and 10 MPa: (**a**) 30 min; (**b**) 60 min; (**c**) 90 min.

Figure 8 shows the microstructure of the bonding interface at different holding times (30 min, 60 min, 90 min) under the conditions of a 10 μm foil thickness, 750 °C, and 10 MPa. The white dotted lines in the figure represent the SPS diffusion-bonded interfaces. As the holding time increases from 30 min to 90 min, the voids at the bonding interface transition from a continuous to a more dispersed distribution, and gradually disappear. At 90 min, the voids are completely closed. This improvement is attributed to the increased atomic diffusion between the interlayer and the base material at the interface, which becomes more sufficient with the prolonged holding time, leading to a more complete interfacial reaction and a stronger joint.

Table 4 shows the shear strength and deformation rate at different holding times. Both the shear strength and deformation rate increase with the extension of the holding time. As the holding time increases from 30 min to 90 min, the shear strength increases from 95.45 MPa to 119.62 MPa. This improvement is mainly attributed to the extended holding time, which allows more time for atomic diffusion between the interlayer and the base materials on both sides of the bonding interface. As the diffusion process becomes more complete, the interfacial bonding quality improves, leading to a reduction in defects and an increase in the shear strength of the joint.

### 3.4. Effect of Bonding Pressure

**Figure 9:**
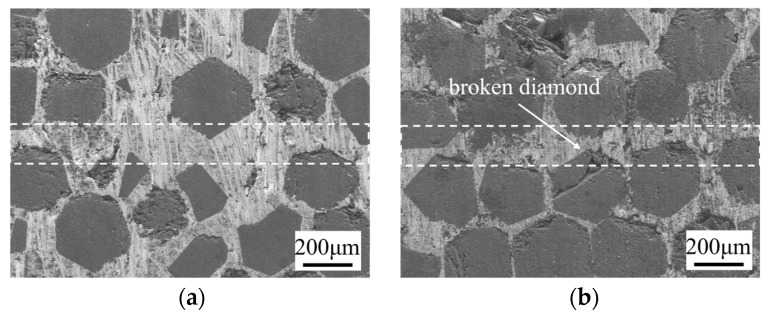
Microstructure of bonding interface at different bonding pressures under 10 μm, 750 °C, and 60 min: (**a**) 7.5 MPa; (**b**) 12 MPa.

Figure 9 shows the microstructure of the bonding interface under different pressures (7.5 MPa, 10 MPa, 12.5 MPa) at 10 μm, 750 °C, and 60 min. With increasing pressure, the density of diamond at the interface increases. The white dotted lines in the figure represent the SPS diffusion-bonded interfaces. At 12.5 MPa, some diamond particles exhibit visible fractures, which is attributed to the increased mechanical compression that occurs when diamonds on both sides are pressed into close proximity. Such fractures compromise the integrity of the diamond and may adversely affect the TC of the diamond/Cu composite [30,31]. The mechanical properties of the joints under different bonding pressures are shown in Table 5. As the pressure increases from 7.5 MPa to 12.5 MPa, the shear strength of the joint increases from 97.89 MPa to 116.37 MPa, and the deformation rate increases from 0.62% to 1.25%.

## 4. Thermal Conductivity of the Diffusion-Bonded Interface

Considering the bonding quality of the joint, it was found that the bonding interface was free of defects, and the highest shear strength was achieved under the conditions of 10 μm, 810 °C, 60 min, and 10 MPa. At this time, the TC of the joint reached 700.97 W/(m·K), which is 85.62% of that of the base metal. Hu et al. [3] found that the TC of diamond/Cu composites without an interlayer after SPS diffusion bonding was 347.73 W/(m·K), which is 62.21% of that of the base metal. Compared to direct bonding without an interlayer, diffusion bonding with a Cr interlayer exhibits better TC.

The improvement in TC can be attributed to two main factors. First, the interlayer helps reduce cracks and voids at the bonding interface, thereby lowering the interfacial thermal resistance. Second, Cr reacts with C in diamond to form Cr_3_C_2_. Cr_3_C_2_ can improve the interfacial bonding between Cu and diamond and regulates the acoustic mismatch between Cu and diamond, which contributes to the enhanced TC of the diffusion-bonded interface [32,33,34].

## 5. Conclusions

This study investigates the SPS diffusion bonding of diamond/Cu composites, focusing on the effects of interlayer thickness, bonding temperature, holding time, and bonding pressure on their microstructure and properties. The main conclusions are as follows:

(1) At an interlayer thickness of 10 μm, microcracks, and voids are observed at the bonding interface. At 30 μm, no significant defects are found, and the shear strength reaches 131.27 MPa. However, when the interlayer thickness increases to 50 μm, the shear strength drops to 63.60 MPa, and discontinuous voids appear at the bonding interface.
(2) Increasing the bonding temperature, holding time, and bonding pressure improves mechanical properties and reduces defects at the bonding interface. The higher temperature and pressure promote atomic diffusion, improving bonding quality and minimizing defects. Additionally, the extended holding time allows for more diffusion, further enhancing joint formation.
(3) Under the conditions of a 10 μm interlayer, 810 °C, 60 min, and 10 MPa, the bonding interface achieves the most compact structure, with a TC of 700.97 W/(m·K), a shear strength of 139.89 MPa, and a deformation rate of just 1.09%.

## Figures and Tables

**Table 1:** The experimental scheme for SPS diffusion bonding.

No.	Temperature/°C	Holding Time/min	Pressure/MPa	Interlayer Thickness/μm
1	720	60	10.0	10
2	750	60	10.0	10
3	780	60	10.0	10
4	810	60	10.0	10
5	750	30	10.0	10
6	750	90	10.0	10
7	750	60	7.5	10
8	750	60	12.5	10
9	750	60	10.0	30
10	750	60	10.0	50

**Table 2:** Mechanical properties of joints with different interlayer thicknesses.

Interlayer Thickness/μm	Shear Strength/MPa	Deformation Rate/%
10	108.97	0.78
30	131.27	1.03
50	63.60	1.16

**Table 3:** Mechanical properties of joints with different bonding temperatures.

Bonding Temperature/°C	Shear Strength/MPa	Deformation Rate/%
720	71.38	0.52
750	108.97	0.78
780	115.59	1.03
810	139.89	1.09

**Table 4:** Mechanical properties of joints with different holding times.

Holding Time/min	Shear Strength/MPa	Deformation Rate/%
30	95.45	0.52
60	108.97	0.78
90	119.62	1.20

**Table 5:** Mechanical properties of joints with different bonding pressures.

Bonding Pressures/MPa	Shear Strength/MPa	Deformation Rate/%
7.5	97.89	0.62
10	108.97	0.78
12.5	116.37	1.25

## Data Availability

The data are contained within the article.
